# Green materials science and engineering reduces biofouling: approaches for medical and membrane-based technologies

**DOI:** 10.3389/fmicb.2015.00196

**Published:** 2015-03-17

**Authors:** Kerianne M. Dobosz, Kristopher W. Kolewe, Jessica D. Schiffman

**Affiliations:** Department of Chemical Engineering, University of Massachusetts AmherstAmherst, MA, USA

**Keywords:** antibiotic resistance, antifouling, biofouling, green chemistry, resistance genes, drug development

## Abstract

Numerous engineered and natural environments suffer deleterious effects from biofouling and/or biofilm formation. For instance, bacterial contamination on biomedical devices pose serious health concerns. In membrane-based technologies, such as desalination and wastewater reuse, biofouling decreases membrane lifetime, and increases the energy required to produce clean water. Traditionally, approaches have combatted bacteria using bactericidal agents. However, due to globalization, a decline in antibiotic discovery, and the widespread resistance of microbes to many commercial antibiotics and metallic nanoparticles, new materials, and approaches to reduce biofilm formation are needed. In this mini-review, we cover the recent strategies that have been explored to combat microbial contamination without exerting evolutionary pressure on microorganisms. Renewable feedstocks, relying on structure-property relationships, bioinspired/nature-derived compounds, and green processing methods are discussed. Greener strategies that mitigate biofouling hold great potential to positively impact human health and safety.

## Introduction

Biofilms are communities of aggregated microorganisms surrounded by a self-produced matrix of extracellular polymeric substances. Across industries, including, healthcare, food production, and membrane-based separation processes, biofilms yield detrimental results (Baker and Dudley, 1998; Van Houdt and Michiels, 2010). Within the clinical setting, bacterial colonization, and subsequent biofilm formation is a pressing challenge that leads to chronic infections (Flemming and Wingender, 2010). Foodborne illnesses associated with bacterial contamination during food processing yield enhanced tolerance to antibiotic treatments (da Silva and De Martinis, 2013). Once fouled, the lifetime, and performance of membranes are significantly decreased, which leads to monetary and health ramifications. Prevention of bacteria attachment is the most effective method of preventing disease, reducing operational costs, and saving energy.

In membrane-based technologies, one approach to eliminate biofouling is to attach biocidal nanomaterials, including silver (Mauter et al., 2011), copper (Dasari et al., 2012), selenium (Akar et al., 2013), and titanium dioxide to the surface of a membrane. To inactivate microbes, commercial antibacterial agents have been released from polymer medical devices (Ng et al., 2013). However, these approaches yield concerns related to the antibacterial agent release rate, depletion, and toxicity to human cells (Schiffman and Elimelech, 2011). Furthermore, the widespread resistance of microbes toward antimicrobials underscores the importance of developing alternative strategies that mitigate the initial attachment of bacteria without exerting evolutionary pressure. Ultrafiltration (UF) membrane surface chemistry plays a role in their propensity to foul. Commercial UF membranes are fabricated from inexpensive, hydrophobic polymers—polysulfone (PSf), polyethersulfone, polypropylene, or polyvinylidene chloride. While these membranes provide proper mechanical and chemical stability, they suffer from biofouling.

In this mini-review, we discuss the benefits of engineering biopolymers and cover recent strategies from medical and membrane-based technologies that have been reported to combat microbial contamination with less evolutionary pressure on microorganisms, meaning that bacteria have shown less resistance to these greener approaches. Biopolymers, surface topography, nature-derived antimicrobials, and green processing are discussed. These green strategies hold great potential to positively impact human health and safety.

## Starting With Greener Polymers

Biopolymers are polymers derived from naturally occurring matter such as crustacean shells, mushrooms, or wood. In addition to being sustainable, biopolymers also offer inherent properties such as, antibacterial activity, biodegradability, biocompatibility, chelation, and coagulation capabilities (Schiffman and Schauer, 2008). One example is chitin and its deacetylated derivative chitosan, which have been heavily investigated for wound healing scaffolds due to their biocompatibility and cationic amine groups, which provide antibacterial activity (Kong et al., 2010). However, working with biopolymers can introduce complications. Chitin can be extracted from a wide number of natural sources including crustacean shells, insect cuticles, and fungal biomass (Schiffman and Schauer, 2009; Hajji et al., 2014). Based on the source, the extracted chitin will vary in molecular weight, degree of deacetylation, purity, distribution of charged groups, and crystallinity. While natural variability can complicate controlled manufacturing, the intrinsic benefits cannot be overlooked. For this reason biopolymers derived from natural feedstocks including, chitin, pectin, cellulose, gelatin, and alginate, have been investigated for biomedical and environmental technologies (Lee and Mooney, 2012; Kalia et al., 2013; Birch and Schiffman, 2014).

## Green Materials Science and Engineering for Biomedical Applications

As noted previously, biopolymers offer intrinsic functionality and biocompatibility making them ideal hydrogel tissue engineering scaffolds (Van Vlierberghe et al., 2011). Biodegradable polymers, including, polylactic acid, polycaprolactone, and poly-alhyl-cyanoacrylates are used for temporary therapeutics and drug delivery vehicles that limit biofouling, while maintaining biocompatibility (Kumari et al., 2010). Numerous other review articles discuss polymers for biomedical implants, here we focus on alternative strategies that could potentially be used synergistically with polymeric medical devices to decrease bacterial contamination.

### Greener Antifouling and Antibacterial Surfaces

Antimicrobial materials kill microbes through passive contact with functionalized cationic/biomolecule groups or via interactions with released antimicrobial compounds (Isquith et al., 1972; Ouattara et al., 2000). In an effort to move away from antimicrobials that cause evolutionary pressure on microorganisms, the targeting specificity of cationic peptides have demonstrated excellent potential in disrupting biofilms (Hofmann et al., 2012). Plant derivatives are ideal candidates for active antibacterial agents (Burt, 2004). Due to the polydispersity of essential oils – carvacrol, cinnamaldehyde (Zodrow et al., 2012), green tea (Reygaert, 2014) – they do not exhibit bacterial resistance. The small volatile molecules have been delivered via carrier-solutions, polymer derivatives, or encapsulated in solid particles/films (Kavanaugh and Ribbeck, 2012; Zodrow et al., 2012; Badawy and Rabea, 2013; Carbone-Howell et al., 2015; Rieger et al., 2015). Recently, we have demonstrated the ability to incorporate essential oils into biopolymer nanofiber mats (Rieger and Schiffman, 2014) and ultra-thin films (Rieger et al., 2015). In time dependent cytotoxicity studies on the biopolymer nanofibers, the intrinsic antibacterial activity of chitosan along with the quick release of cinnamaldehyde from the nanofibers enabled high inactivation rates against *Escherichia coli* and *Pseudomonas aeruginosa* (Rieger and Schiffman, 2014).

Antifouling surfaces prevent the adhesion of microbes and proteins to surfaces via super hydrophobic or hydrophilic properties (Keefe et al., 2012). Polyethylene glycol (PEG) is a preeminent polymer for biomedical applications (Langer and Tirrell, 2004) because the biocompatible polymer forms a hydration layer with the surrounding environment to provide non-specific antifouling ability. However, PEG-based materials oxidize after exposure to physiological environments, thus limiting their long term effectiveness. Another class of non-fouling polymers that have a broader chemical diversity are zwitterionic polymers, which offer positive and negative charges on a single monomer (polybetaines), or different monomers (polyampholytes) (Chen et al., 2010).

### Topographic Cues and Substrate Stiffness Influence Microbial Behavior

Surface topography has been proposed as a non-toxic surface modification to reduce bacterial adhesion (Hoffman, 2002; Engel et al., 2012; Rizzello et al., 2013; Harding and Reynolds, 2014). **Table 1** summarizes recent investigations into the effect that microscale topography has on biofilm development. Nanotopographic patterning or biomimetic surfaces can also limit bacterial adhesion (Scardino and de Nys, 2011). For example, independent of feature dimensions (square, rectangular, or circular posts), it was reported (Perera-Costa et al., 2014) that organized topography significantly reduces bacterial attachment. Engineered roughness index has been proposed as a possible explanation for the reduction of microbial adhesion, however, the general mechanism remains poorly understood (Bazaka et al., 2011).

**Table 1:** **Microorganisms respond to surface topography. Schematics of the topographies are provided, as well as highlighted examples with figures reprinted (adapted) with permission from the American Chemical Society. The dimensions given include length (l), width (w), height (h), diameter (d), and interspatial spacing (s). All substrates are polydimethylsiloxane (PDMS) except for the parallel fibers**.

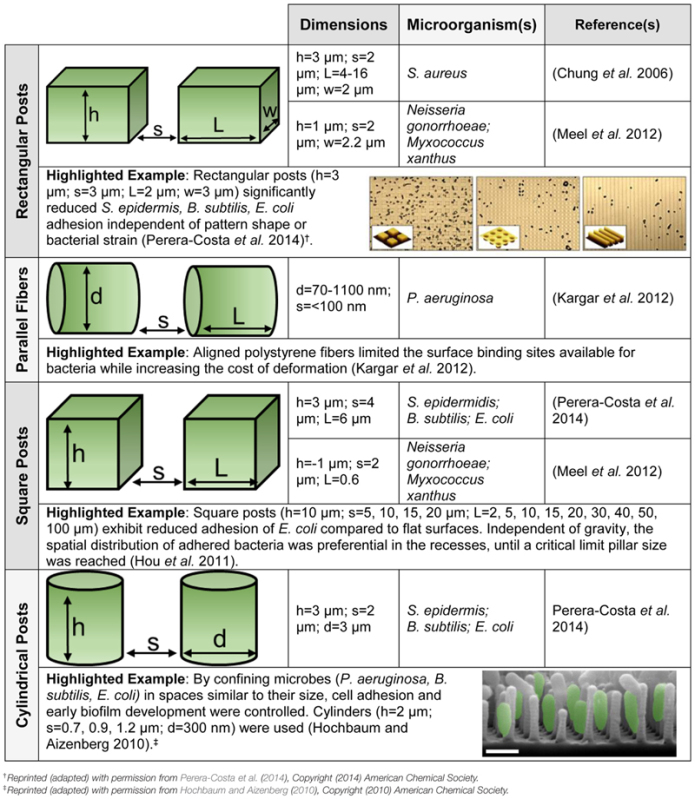

Substrate stiffness is a tunable material property that limits bacterial adhesion without inducing resistance development (Lichter et al., 2008). Effective stiffness was utilized in a polydimethylsiloxane (PDMS) nanoarray to control the spatial organization of *P. aeruginosa* around compliant nanoposts (Epstein et al., 2011).

## Green Materials Science and Engineering for Membrane-based Technologies

### Bio-inspired Membrane Modifications Reduce Biofouling

**FIGURE 1:**
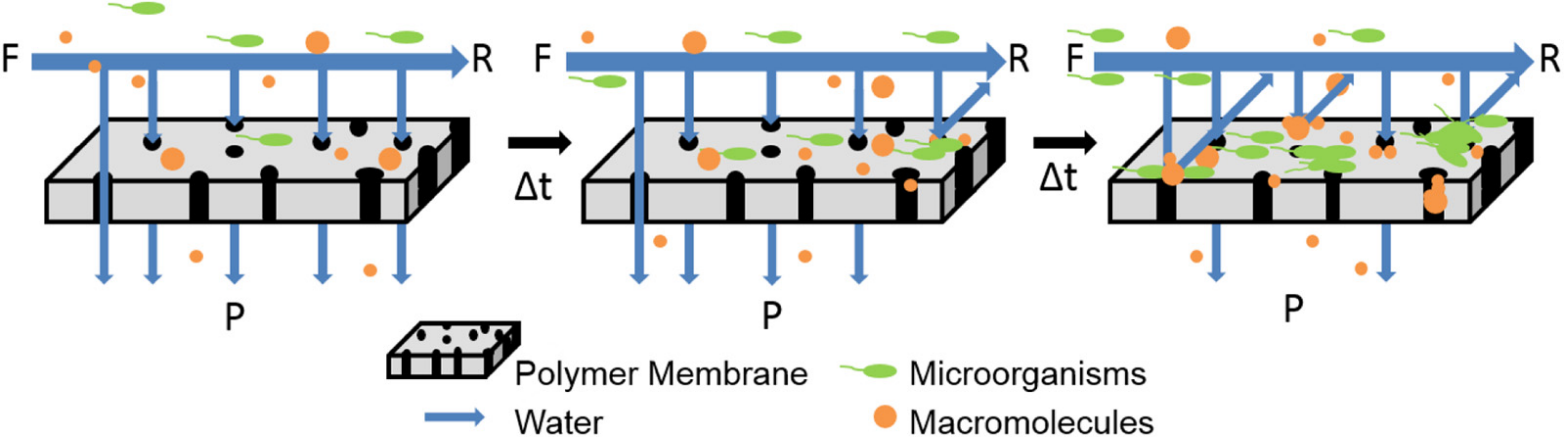
**Schematic illustrates that membranes become fouled when operated in cross-flow systems with constant flow and pressure.** Direction of feed (F), retentate (R), and permeate (P) are provided.

During the standard membrane operation, microbes, or macromolecules in the feed solution components accumulate on the surface of the membrane, which leads to retardation of flux and loss of performance, as shown in **Figure 1**. **Table 2** provides a schematic of this phenomena called biofouling, as well as highlights green strategies to minimize biofouling. Previous reviews have covered the traditional approaches employed to reduce biofouling on membranes, including the use of synthetic polymers and metallic ions (Baker and Dudley, 1998; Kumar and Anand, 1998; Mansouri et al., 2010; Perera et al., 2014). In general, the membrane field could also look toward greener approaches being tested in the medical and food industries to combat biofouling (Simoes et al., 2010; Cappitelli et al., 2014).

**Table 2:** **This table highlights recent publications that have investigated green modifications to microfiltration, ultrafiltration, and reverse osmosis membranes**.

Modification(s) and Membrane	Effect(s)	Reference
Capsaicin derivatives blended into membrane body or surface modification on UF PSF membranes.	Increased water flux in blended membrane, increased flux, antifouling and antibacterial properties when challenged by humic acid.	Xu et al. (2013), Wang et al. (2014)
*N*-succinyl or *N*-propylphosphonic chitosan blended into UF PSf membranes.	Increased hydrophilicity, flux, and fouling resistance ratio when challenged with bovine serum albumin (BSA).	Kumar et al. (2013a,b)
δ-Gluconolactone surface modification on chloromethylated UF PSf membranes.	Increased human serum albumin rejection from 84 ± 1% to 96 ± 1%. Increased pure water flux resistance by 14%.	Fan et al. (2012)
Lysozyme surface modification on PA RO membranes.	Increased water flux resistance, antibacterial activity against Gram-positive bacteria, and antifouling properties.	Saeki et al. (2013)
Myoglobin surface modification on UF polyethersulfone membranes.	Increased hydrophilicity and increased lysozyme rejection by up to 21.43%.	Ali and Tari (2012)
PDA surface modification on MF PVDF membranes.	Increased organic rejection. Flux persisted from pristine to modified membrane.	McCloskey et al. (2010, 2012)
PDA, PDA-graft-PEG, and PDA co-polymers surface modification on UF PSf membranes.	Increased antifouling efficiency and increased flux transmembrane pressure when challenged with soybean emulsions, BSA, and oil.	Choi et al. (2014), Miller et al. (2014)
PDA surface modification on thin-film composite RO membranes.	Increased pure water resistance with increasing PDA. Increased flux during oil/water separations.	Kasemset et al. (2013)

*Abbreviations: Microfiltration (MF), Polyamide (PA), Polydopamine (PDA), Polyethylene glycol (PEG), Polysulfone (PSf), Poly(vinylidene fluoride) (PVDF), Reverse osmosis (RO), Ultrafiltration (UF).*

Numerous recent reports have explored the use of biopolymers to reduce biofouling in membrane-based separations. Cellulose acetate nanofiltration membranes surface modified with sodium alginate and chitosan showed a 15% flux increase when challenged with bovine serum albumin (BSA) (Lajimi et al., 2011). Higher permeability was achieved by blending *N*-succinyl chitosan into UF membranes (Kumar et al., 2013a). Membranes with *N*-propylphosphonic chitosan added to their surface exhibited higher permeability and antifouling properties over pristine PSf membranes (Kumar et al., 2013b). Exploring advantageous charges, *N*-carboxymethyl chitosan and *O*-carboxymethyl chitosan-based amphoteric or pH responsive charged membranes were prepared for protein separation. It was reported that even after 50-days of operation in a protein environment there was no membrane fouling (Chakrabarty and Shahi, 2014). Recently, the addition of layer-by-layer films of chitosan and carboxymethyl cellulose to partially deacetylated cellulose acetate films yielded a 55% reduction in BSA adsorption (Mohan et al., 2015).

Polydopamine (PDA) is a bio-inspired polymer that mimics the adhesion secretions of mussels (Brubaker and Messersmith, 2012). The self-polymerizing polymer is capable of anchoring to and protecting surfaces from microbial contamination (Lee et al., 2007; Dreyer et al., 2012). PSf UF membranes and commercial polyamide (PA) reverse osmosis (RO) membranes modified with PDA exhibited an increase in antifouling properties (Kasemset et al., 2013; Miller et al., 2014). Additional research with PSf UF membranes includes coating the membranes with dopamine methacrylamide and a plant-based methacrylate, which showed higher biofouling resistance and bactericidal properties than the control membranes (Choi et al., 2014). Due to their larger pore size, flux was maintained after coating PDA on poly(vinylidene fluoride, PVDF) microfiltration (MF) membranes, as opposed to a similar modification conducted on RO and UF membranes (McCloskey et al., 2010). PDA coatings have increased the rejection during oil/water emulsion separations (McCloskey et al., 2012) and improved the mechanical properties and hydrophilicity of electrospun nanofiber membranes for filtration applications (Huang et al., 2014).

Biological molecules have also been explored to improve membrane properties. This includes attaching serine protease to the surface of cellulose acetate UF membranes, which resulted in a relative flux reduction ratio of 97–88%, along with an increase in steady state flux from 8 to 34 L m^-2^h^-1^ for the pristine and treated membranes, respectively (Koseoglu-Imer, 2013). Polyethersulfone UF membranes surface modified with myoglobin increased membrane hydrophilicity by 47.13% and lysozome rejection by 21.43% (Ali and Tari, 2012). The surface of chloromethylated PSf membranes modified with gluconolactone had improved anti-protein adsorption ability (Fan et al., 2012). The body of PSf membranes were blended with a ternary copolymer having capsaicin-mimic moieties improved the permeate flux and rejection when challenged by a humic acid solution and a seawater solution; excellent antibacterial efficiency was also reported (Xu et al., 2013). Capsacin grafted to the surface PSf membranes demonstrated improved antifouling and antibacterial properties (Wang et al., 2014). RO membranes surface modified with lysozyme showed sufficient antibacterial activity against the Gram-positive bacteria, *Micrococcus lysodeikticus,* and *Bacillus subtilis* (Saeki et al., 2013). When heparin was attached to the surface of chitosan/cellulose acetate membranes they demonstrated antifouling characteristics, but not antibiofouling (Liu et al., 2010). The essential oil, cinnamaldehyde, was released for ∼2 days whereas kanamycin was released for ∼80 h from the surface of RO membranes via biodegradable poly (lactic-co*-*glycolic acid) particles (Zodrow et al., 2014). However, a significant reduction in biofilm development was only observed on membranes modified with kanamycin capsules. Smaller size molecules, acids have been incorporated into membranes. By adsorbing citric acid onto the surface of UF PSf membranes, PEG rejection, BSA rejection, and flux recovery ratios increased (Wei et al., 2012). PSf membranes with the addition of ascorbic acid, citric acid, and malic acid into the body of membrane reported a superior pure water flux and higher permeation and rejection compared to control membranes (Ghaemi et al., 2012).

### Greener Solvents can Improve Membrane Properties

Improvements to the membrane fabrication process have recycled and reduced the amount of noxious and waste solvent. In an effort to replace the flammable, toxic, and teratogenic membrane-casting solvents, dimethylformamide, and dimethyl sulfoxide, the use of non-toxic, non-flammable, and inexpensive supercritical carbon dioxide has been investigated (Barroso et al., 2011). Polyacrylonitirile graft polyethylene oxide membranes cast using supercritical carbon dioxide exhibited an increase in hydrophilicity, larger protein/starch permeability, and an increased resistance to fouling (Barroso et al., 2011). Additionally, antifouling membranes have been synthesized using a solvent-free approach wherein 2-hydroxyethyl methacrylate was bulk polymerized. The homogenous membranes rejected 97 and 99% of yeast and oil, respectively, (Peng et al., 2013). The easily recoverable ionic liquid 1-ethyl-3-methylimidazolium acetate was used to produce cellulose and chitin active layers. When the bioactive coatings were applied to the surface of electrospun non-woven substrates, a similar rejection paired with a 10-fold increase in permeation flux was reported in comparison to commercial UF membranes (Ma et al., 2011).

## Perspective

Bacteria colonization and biofilm formation are pressing challenges that yield infections, higher energy consumption, and subsequent costs. New, innovative, and green solutions that mitigate these detrimental effects in medical and membrane-based technologies without exerting evolutionary pressure on microbes or on our environment are needed. The intrinsic properties of historically employed biopolymers, naturally derived antimicrobials, and bio-inspired agents can improve the surface hydrophilicity, protein adhesion resistance, and antibacterial activity of materials. However, the long-term viability of surfaces that have been modified with chemical antimicrobials is often limited by microbial and solution surface conditioning. Namely, ions and proteins adsorb onto the surface and mask the surface activity (Palmer et al., 2007). Perhaps an “even greener” method than using biopolymers to create an antifouling surfaces is to avoid chemicals and employ a structure-property relationship.

While organized topography certainly influences microbial behavior, virtually all examples from literature use PDMS. Further effort is needed to elucidate whether structure is a universal effect across all hard and soft surfaces. Incorporating spatially organized topography to medical implants and membranes, potentially, can be synergistically employed with non-specific antimicrobial compounds to extend surface functionality. With economy of scale, many of the same approaches employed to decrease biofouling on high-value biomedical devices may be appropriate for separation membranes. In the future, green materials science and engineering strategies that mitigate biofouling will allow us to overcome current challenges to positively impact human health.

## Conflict of Interest Statement

The Review Editor, James F. Holden, declares that, despite being affiliated with the same institution as authors Kerianne M. Dobosz, Kristopher W. Kolewe, Jessica D. Schiffman, the review process was handled objectively and no conflict of interest exists. The authors declare that the research was conducted in the absence of any commercial or financial relationships that could be construed as a potential conflict of interest.
